# Challenges for 2022 and beyond

**DOI:** 10.4102/sajp.v78i1.1828

**Published:** 2022-12-12

**Authors:** Aimee V. Stewart

**Affiliations:** 1 Department of Physiotherapy, School of Therapeutic Sciences, Faculty of Health Sciences, University of the Witwatersrand, Johannesburg, South Africa

We have come to the end of yet another year – a cliché I know. So how has this year treated us? We are out of the lockdowns imposed at various times and with various levels of severity because of the coronavirus disease 2019 (COVID-19) pandemic. We are slowly picking up the pieces of our previous lives with some changes because of the lockdowns which will undoubtedly stay! Schools and universities are largely back to face-to-face teaching, and many people have returned to their physical workplaces. All in all, an easier year on many levels notwithstanding the many difficulties the world is currently facing. While many health care professionals were focused on and totally involved in the COVID-19 pandemic, other health care challenges continued.

One of these challenges is the realistic management of people with chronic diseases and long-term disability. Physiotherapists are closely involved in the management of both these conditions and constantly need to adjust their management to the current situation and current thinking.

What do our researchers and clinicians need to think about to manage these conditions as we move further into the 21st century? In countries like South Africa, we should increasingly consider how we can encourage our patients with chronic conditions and disabilities to manage themselves. This self-management is important given the numbers of patients that we have, balanced against our constrained resources. Patients should not be expected to find their own information but should be supported by the health care team in a way that takes into consideration their specific needs and circumstances. This can only be done in programmes that consider the individuals’ abilities to manage their own circumstances, and that take into consideration their own abilities, their specific socio-economic situations, their cultural backgrounds as well as their levels of family and community support.

We constantly need to think of our patients within the continuum of managing their impairments, developing their abilities in their required daily activities and finally ensuring their participation as far as possible at all their required levels within their communities. This management approach needs to include all relevant members of the health care team in a holistic management programme centred on the individual needs of our patients within their family structures and communities.

The above would be so much easier if we did not also have the challenge of large numbers of patients relative to the number of qualified physiotherapists in the country. So, we also need to be innovative in the way in which we meaningfully manage patients with chronic diseases and long-term disability. How we can incorporate tele-medicine, appropriate home programmes with regular tailored support from the health care team, caregiver and family education and group exercise programmes close to the places where patients live, are interventions that need increasing consideration and research to make them important mainstream interventions. By considering the measurement of these interventions in well-designed studies, we will be able to introduce those that are appropriate for our patients. In this way we will become innovative and ensure our relevance in the management of the health care needs of our country. These studies should be published and then disseminated and translated to the clinical situation.

Our journal continues to thrive and has continued to publish increasingly good articles. The quality of our submissions has improved over the years as authors see the value of publishing in the *South African Journal of Physiotherapy* (SAJP). We have had articles published from the following countries this year: Belgium, Burundi, Germany/Indonesia (authors affiliated in both these countries), Ghana, Kenya, Taiwan and South Africa. This is very satisfying, and we wish to increase our authorship from more middle- to low-income countries given the relevance and importance of their experiences to our own. Our readership has also increased and now we have readers from most continents, and we have increasing numbers of reviewers from outside South Africa. These reviewers have added to the quality of our review team. All three of these developments are enabling us to be a more international journal. In addition, our citation-based measures are improving all the time and the latest measures can be seen on our SAJP webpage under ‘journal information’.

The above good news would not have occurred if it were not for our dedicated reviewers who give their time so generously and who are increasingly senior and experienced, the publishers who go the extra mile always and our authors who choose to publish with us. The ongoing sponsorship of the SASP is vital to the viability of the journal and we would not be able to function without it.

We expect that this upward trend continues and that we become increasingly one of the journals of choice for physiotherapy researchers and clinicians in middle- and low-income countries. This is where the real challenges are, and which we need to meet to ensure the relevance of our profession within these countries. We also enjoy getting articles from high-income countries obviously, and hope that more authors from them submit articles!

